# TrkB reduction exacerbates Alzheimer's disease-like signaling aberrations and memory deficits without affecting β-amyloidosis in 5XFAD mice

**DOI:** 10.1038/tp.2015.55

**Published:** 2015-05-05

**Authors:** L Devi, M Ohno

**Affiliations:** 1 Center for Dementia Research, Nathan Kline Institute, Orangeburg, NY, USA; 2 Department of Psychiatry, New York University Langone Medical Center, New York, NY, USA

## Abstract

Accumulating evidence shows that brain-derived neurotrophic factor (BDNF) and its receptor tropomyosin-related kinase B (TrkB) significantly decrease early in Alzheimer's disease (AD). However, it remains unclear whether BDNF/TrkB reductions may be mechanistically involved in the pathogenesis of AD. To address this question, we generated 5XFAD transgenic mice with heterozygous TrkB knockout (TrkB^+/–^·5XFAD), and tested the effects of TrkB reduction on AD-like features in this mouse model during an incipient stage that shows only modest amyloid-β (Aβ) pathology and retains normal mnemonic function. TrkB^+/–^ reduction exacerbated memory declines in 5XFAD mice at 4–5 months of age as assessed by the hippocampus-dependent spontaneous alternation Y-maze task, while the memory performance was not affected in TrkB^+/–^ mice. Meanwhile, TrkB^+/–^·5XFAD mice were normal in nest building, a widely used measure for social behavior, suggesting the memory-specific aggravation of AD-associated behavioral impairments. We found no difference between TrkB^+/–^·5XFAD and 5XFAD control mice in cerebral plaque loads, Aβ concentrations including total Aβ42 and soluble oligomers and β-amyloidogenic processing of amyloid precursor protein. Interestingly, reductions in hippocampal expression of AMPA/NMDA glutamate receptor subunits as well as impaired signaling pathways downstream to TrkB such as CREB (cAMP response element-binding protein) and Akt/GSK-3β (glycogen synthase kinase-3β) were observed in TrkB^+/–^·5XFAD mice but not in 5XFAD mice. Among these signaling aberrations, only Akt/GSK-3β dysfunction occurred in TrkB^+/–^ mice, while others were synergistic consequences between TrkB reduction and subthreshold levels of Aβ in TrkB^+/–^·5XFAD mice. Collectively, our results indicate that reduced TrkB does not affect β-amyloidosis but exacerbates the manifestation of hippocampal mnemonic and signaling dysfunctions in early AD.

## Introduction

Brain-derived neurotrophic factor (BDNF) and its receptor tropomyosin-related kinase B (TrkB) decline in brains of mild cognitive impairment or early Alzheimer's disease (AD).^1, 2^ As BDNF/TrkB signaling is responsible for neuronal growth, protection and plasticity,^3, 4^ its dysfunction as a consequence of amyloid-β (Aβ) accumulation in brain is hypothesized to underlie synaptic and cognitive impairments associated with AD.^5, 6, 7^ In accordance with this hypothesis, central BDNF administration or gene delivery in rodent and primate models of AD produces beneficial effects against Aβ-induced neurotoxicity, synapse loss, aberrant gene expression, deficient cell signaling and impairments in learning and memory.^8, 9^ Furthermore, recent work from our laboratory and others demonstrates that systemic administration of 7,8-dihydroxyflavone (7,8-DHF), a small-molecule TrkB agonist, in amyloid precursor protein (APP) transgenic mice improves their synaptic loss and dysfunction, and memory deficits in a battery of hippocampus-dependent paradigms,^10, 11, 12, 13^ representing a feasible therapeutic approach that is more efficacious and far less invasive than viral or recombinant BDNF delivery.

Meanwhile, there is also experimental evidence suggesting that BDNF/TrkB signaling may be an important regulator of amyloidogenic processing. For example, Aβ production in cultured cells is reduced by BDNF treatment,^14^ while it is facilitated by BDNF deprivation.^15^ Chronic activation of TrkB receptors with 7,8-DHF is reported to exert beneficial effects in APP mice, at least in part, by alleviating cerebral Aβ accumulation.^10, 11^ The pathogenic role of deficient BDNF/TrkB signaling is also supported by the observations that environmental risks for sporadic AD (for example, aging, stress, high-fat diet and so on) impair BDNF/TrkB pathways,^16, 17, 18^ whereas factors that may reduce AD risks (for example, exercise, environmental enrichment, social interaction, dietary restriction and so on) elevate BDNF/TrkB levels.^13, 19, 20, 21, 22, 23^ Moreover, accumulating data suggest that BDNF polymorphisms (for example, Val66Met, Cys270Thr and so on) may be associated with an increased risk of developing AD.^5, 24, 25^ In view of these findings, the present study was undertaken to rigorously determine the mechanisms by which deficient BDNF/TrkB signaling may be responsible for inducing or accelerating AD and related cognitive impairments. Specifically, after crossing TrkB^+/–^ knockout and 5XFAD transgenic mice, we examined the effects of TrkB haploinsufficiency (that is, 50% reduction) on the development of AD-like phenotypes, including cognitive impairments, Aβ accumulation and aberrant hippocampal signaling pathways, in young 5XFAD mice that show only modest plaque pathology and still retain normal learning and memory function.

## Materials and methods

### Animals

We used 5XFAD mice (Tg6799 line) that co-overexpress familial AD (FAD) mutant forms of human APP (Swedish mutation: K670N and M671L; Florida mutation: I716V and London mutation: V717I) and presenilin 1 (PS1; M146L and L286V mutations) transgenes under transcriptional control of the neuron-specific Thy-1 promoter.^26, 27, 28^ 5XFAD line was maintained by crossing hemizygous transgenic mice with C57Bl/6 breeders (Taconic, Hudson, NY, USA). To compare changes in nesting behavior in different age groups of 5XFAD mice, 5XFAD hemizygotes were used for the experiment with non-transgenic wild-type littermate mice served as controls. For the study of the TrkB agonist 7,8-DHF, 5XFAD transgenic and wild-type mice at 12 months of age received repeated intraperitoneal administration of 5 mg kg^−1^ 7,8-DHF or 17% dimethyl sulfoxide vehicle once daily for 10 consecutive days, as described previously.^10^

Although TrkB homozygous knockout mice die by 2–3 weeks of age, heterozygous mice survive normally into adulthood.^29, 30^ TrkB^+/–^ knockout mice were used as an animal model to recapitulate partial reduction of TrkB expression observed in mild cognitive impairment or incipient AD brains.^1^ For our study, hemizygous 5XFAD transgenic mice were crossbred to TrkB^+/–^ mice (B6/129S2 background)^31^ (stock number: 002544, Jackson Laboratory, Bar Harbor, ME, USA), yielding animals with four different genotypes (wild type, TrkB^+/–^, 5XFAD^+/–^ and TrkB^+/–^·5XFAD^+/–^). Genotyping was performed by PCR analysis of tail DNA and all experiments were done blind with respect to the genotype of mice. Procedures were conducted in accordance with the National Institutes of Health *Guide for the Care and Use of Laboratory Animals* and approved by the Nathan Kline Institute Animal Care and Use Committee.

### Behavioral tests

The mice were tested for the spontaneous alternation Y-maze and nest-building tasks. After behavioral testing, some mice were killed for immunoblotting and enzyme-linked immunosorbent assay (ELISA) experiments, and others were perfused for immunohistochemistry.

### Spontaneous alternation Y-maze test

Spontaneous alternation performance was tested using a symmetrical Y-maze, as described previously.^32, 33^ Each mouse was placed in the center of the Y-maze and was allowed to explore freely through the maze during an 8-min session. The sequence and total number of arms entered were recorded. Arm entry was considered to be complete when the hind paws of the mouse had been completely placed in the arm. Percentage alternation is the number of triads containing entries into all three arms divided by the maximum possible alternations (the total number of arms entered minus 2) × 100. As the re-entry into the same arm was not counted for analysis, the chance performance level in this task was 50% in the choice between the arm mice visited more recently (non-alternation) and the other arm they visited less recently (alternation).

### Nest building

Nest-building behavior of mice was tested as described previously.^34, 35^ Mice were individually housed for at least 24 h in clean plastic cages with ~1 cm of corn cob bedding lining the floor and identification cards coded to render the experimenter blind to the sex and genotype of each mouse. Two hours before the onset of the dark phase of the lighting cycle, each cage was supplied a commercially available compressed cotton square (Nestlet, 5 × 5 cm, Ancare, Bellmore, NY, USA). The next morning (~16 h later), cages were inspected for nest construction. Pictures were taken before evaluation for documentation. Nest construction was scored using the established system of Deacon with a 5-point scale.^35^ In brief, the scores were as follows: (1) Nestlet not noticeably touched (>90% intact); (2) Nestlet partially torn (50–90% remaining intact); (3) Nestlet mostly shredded but often no identifiable nest site; (4) an identifiable but flat nest; (5) a (near) perfect nest with clear crater (please see the protocol by Deacon^35^ for more detailed scoring standard).

### Immunoblot analysis

Hemibrain samples (excluding the cerebellum) or hippocampal samples were taken from the mice under deep isoflurane anesthesia and were snap-frozen for biochemical assays. For western blot analysis, each sample was homogenized in eightfold volumes of cold homogenization medium containing 70 mM sucrose, 210 mM mannitol, 2 mM HEPES, 0.1 mM EDTA and protease phosphatase inhibitor cocktail, and centrifuged at 10 000 *g* for 10 min to remove any insoluble material. Protein concentrations were determined by a BCA protein assay kit (Pierce, Rockford, IL, USA), and 10–50 μg of protein was run on NuPAGE 4–12% or 10% Bis-Tris gels (Invitrogen, Carlsbad, CA, USA) and transferred to nitrocellulose membrane. After blocking, membranes were probed with the following primary antibodies: anti-TrkB (1:1000, ab51190, Abcam, Cambridge, MA, USA), anti-phospho-TrkB (Y705, 1:500, ab52191, Abcam), anti-BACE1 (1:1000, B0681, Sigma-Aldrich, St Louis, MO, USA), an antibody that recognizes C-terminal epitope in APP (1:1000, C1/6.1, kindly provided by Dr Paul Mathews, Nathan Kline Institute) to detect full-length APP/C-terminal fragments, an antibody specific for the β-secretase-cleaved soluble ectodomain of APP (sAPPβ) (1:1000, SIG-39138, Covance, Princeton, NJ, USA), anti-phospho-CREB (cAMP response element-binding protein; Ser133, 1:1000, no. 9198, Cell Signaling Technology, Danvers, MA, USA), anti-CREB (1:2000, no. 9197, Cell Signaling Technology), anti-phospho-Akt (Ser473, 1:1000, no. 9271, Cell Signaling Technology), anti-Akt (1:1000, no. 4685, Cell Signaling Technology), anti-phospho-GSK-3β (glycogen synthase kinase-3β Ser9, 1:1000, no. 9331, Cell Signaling Technology), anti-GSK-3β (1:2000, sc-9166, Santa Cruz Biotechnology, Dallas, TX, USA), anti-GluA1 (1:1000, no. 13185, Cell Signaling Technology), anti-GluA2 (1:1000, no. 5306, Cell Signaling Technology), anti-NR1 (1:1000, no. 5704, Cell Signaling Technology), anti-NR2A (1:2000, no. 4205, Cell Signaling Technology), anti-NR2B (1:2000, no. 4212, Cell Signaling Technology) and anti-β-actin (1:15 000, AC-15, Sigma-Aldrich). The membranes were then incubated with horseradish peroxidase-conjugated secondary IgG. Immunoblot signals were visualized by an ECL chemiluminescence substrate reagent kit (Pierce) and quantified by densitometric scanning and image analysis using Quantity One software (Bio-Rad Laboratories, Hercules, CA, USA).

### ELISAs of soluble Aβ oligomers and total Aβ42

To measure the concentrations of soluble Aβ oligomers, each hemibrain sample was homogenized in eightfold volumes of homogenization medium, as described above. To quantitate total levels of Aβ42, the other hemibrain was extracted in eightfold volumes of cold 5 M guanidine HCl plus 50 mM Tris HCl (pH 8.0) buffer and centrifuged at 20 000 *g* for 1 h at 4 °C to remove insoluble material. Final guanidine HCl concentrations were <0.1 M. Protein concentrations were determined by a BCA protein assay kit (Pierce). Supernatant fractions were analyzed by well-established human Aβ ELISA kits specific to oligomeric forms of Aβ (27725, IBL America, Minneapolis, MN, USA) and Aβ42 (KHB3441, Invitrogen) according to the protocols of the manufacturers. Optical densities at 450 nm of each well were read on a VersaMax tunable microplate reader (Molecular Devices, Sunnyvale, CA, USA), and sample Aβ oligomer and Aβ42 concentrations were determined by comparison with the respective standard curves. Aβ oligomer and Aβ42 concentration values were normalized to total brain protein concentrations and expressed in picograms and nanograms per milligram of total protein, respectively.

### Aβ immunohistochemistry

Mice were transcardially perfused with 0.1 M phosphate-buffered saline (PBS; pH7.4), followed by 4% paraformaldehyde in PBS under deep isoflurane anesthesia. Brains were postfixed for 24 h in 4% paraformaldehyde in PBS at 4 °C and transferred to PBS. The brain was sectioned coronally at 30 μm using a vibratome (VT1200, Leica Microsystems, Wetzlar, Germany), and successive sections were stored in PBS containing 0.05% sodium azide at 4 °C. Two sections per mouse taken at levels between –1.7 and –1.9 mm to bregma according to the mouse brain atlas of Franklin and Paxinos^36^ were stained by the avidin–biotin peroxidase complex method as described previously.^33, 37, 38^ In brief, the sections were incubated overnight at 4 °C with mouse monoclonal anti-Aβ1–16 (6E10) antibody (1:200, SIG-39347, Covance). The avidin–biotin peroxidase complex kit (PK-2200, Vector Laboratories, Burlingame, CA, USA) was utilized with 3,3′-diaminobenzidine tetrahydrochloride as a chromogen to visualize the reaction product. The sections were then mounted on charged slides, dehydrated in a series of alcohol, cleared in xylene and covered with a coverslip. Light microscopy was conducted on an Axioskop 2 microscope equipped with an AxioCaM HRc digital camera (Zeiss, Oberkochen, Germany) for capturing images. Semi-quantitative analysis was performed using AxioVision imaging software with the AutoMeasure module (Zeiss). The threshold optical density that discriminated staining from background was determined and held constant for all quantifications. Identified objects were individually inspected by the same investigator to confirm the object as a plaque or not in a blinded manner. Percentage area occupied by Aβ deposits in the hippocampus and cortex was assessed bilaterally to compare plaque burden between 5XFAD control and TrkB^+/–^·5XFAD mice.

### Statistical analysis

Data were analyzed by a one-way or two-way analysis of variance, and *post hoc* Bonferroni comparisons were performed to determine the significance of differences between the groups when appropriate. Data were presented as mean±s.e.m. and the level of significance was set for *P*<0.05.

## Results

### TrkB reduction exacerbates memory deficits but not impairment of nesting behavior in 5XFAD mice

**Figure 1:**
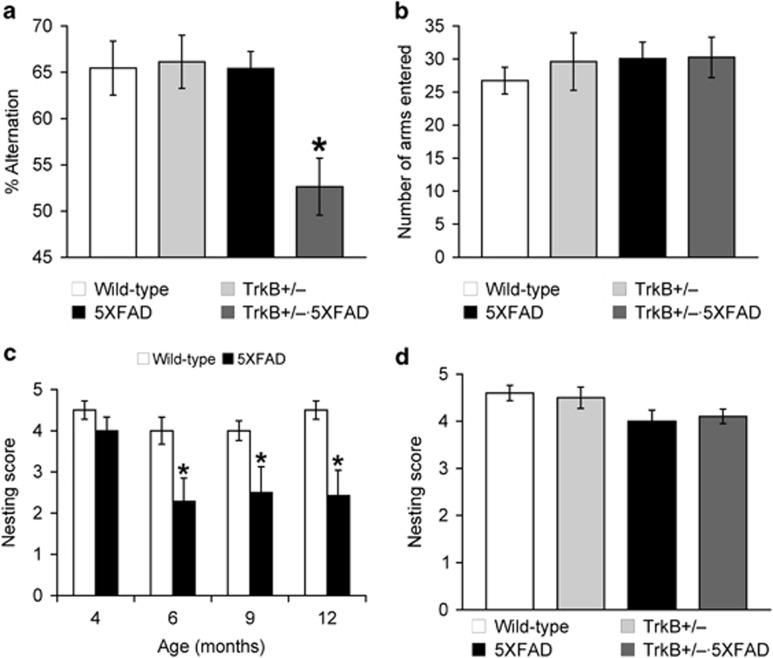
Effects of tropomyosin-related kinase B (TrkB) reduction on memory and nesting behavior in 5XFAD mice. (**a**, **b**) Mice at 4–5 months of age were tested for spatial memory in the Y-maze task (*n*=11–13 mice per group). During 8- min testing, the spontaneous alternation behavior (**a**) and total number of arm entries reflecting exploratory activities (**b**) were measured. Note that TrkB^+/–^·5XFAD mice show significantly lower levels of alternation performance than those of the other three groups of mice (*P<0.05). (**c**) 5XFAD mice show age-dependent declines in nesting score (*P<0.05 vs respective wild-type controls and 4-month-old 5XFAD; *n*=6–15 mice per group). (**d**) TrkB^+/–^ reduction does not exacerbate impairment of nest-building behavior in 5XFAD mice at 4–5 months of age (*n*=6–22 mice per group). All data are presented as mean±s.e.m.

We used 5XFAD mice that represent a rapid onset and aggressive amyloid model based on accelerated Aβ42 production owing to a combination of five FAD mutations in APP and PS1 transgenes.^26, 27, 28^ 5XFAD mice begin to develop visible Aβ deposition as early as 2 months of age^27^ and exhibit memory declines on a battery of hippocampus-dependent tasks around 6 months concomitant with moderate Aβ pathology and synaptic dysfunction at the Schaffer collateral-CA1 pathway.^11, 26, 38, 39, 40, 41, 42, 43, 44^ To test whether TrkB^+/–^ reduction may aggravate AD-associated memory declines in 5XFAD mice, we used the hippocampus-dependent spontaneous alternation Y-maze task (Figure 1a). 5XFAD mice at 4–5 months of age showed spontaneous alternation performance equivalent to that of wild-type controls, retaining normal spatial memory function at this age. Remarkably, levels of spontaneous alternation in TrkB^+/–^·5XFAD mice were reduced to ~50% corresponding to the random performance level in this memory assay (*P*<0.05). Furthermore, the memory performances were indistinguishable between TrkB^+/–^ and wild-type control mice, demonstrating that TrkB haploinsufficiency specifically facilitated memory declines in 5XFAD mice without affecting baseline memory function on the wild-type background. Meanwhile, the total numbers of arm entries reflecting exploratory activities during Y-maze testing were not different between the four groups of mice (Figure 1b), indicating that behavioral alternations in TrkB^+/–^·5XFAD mice were memory-specific.

We next examined whether TrkB^+/–^ reduction may affect nest building, a commonly used measure of social behavior,^35^ in 5XFAD mice. Recent reports demonstrate deficient nesting behavior across different lines of APP-overexpressing transgenic mice such as Tg2576,^34, 45^ APPswe/PS1^46, 47^ and 3xTg-AD.^48^ We found that nest construction behavior was significantly impaired in 5XFAD mice at 6, 9 and 12 months of age (*P*<0.05) but not at 4 months of age, as compared with that of the respective age-matched wild-type controls (Figure 1c). Moreover, as compared with 4-month-old 5XFAD mice, nesting scores were significantly lower in the other three age groups of 5XFAD mice (*P*<0.05). Therefore, 5XFAD mice showed age-dependent deficits in nest-building performance with the onset of 6 months, which was similar to that of hippocampus-dependent memory impairments in this AD model.^38, 39, 40, 41^ Nevertheless, nest building was not affected in TrkB^+/–^·5XFAD mice at 4–5 months of age (Figure 1d), suggesting that TrkB haploinsufficiency specifically aggravated hippocampal memory dysfunction without affecting the impairment of nesting behavior in 5XFAD mice.

### TrkB reduction does not exacerbate β-amyloidosis in 5XFAD mice

**Figure 2:**
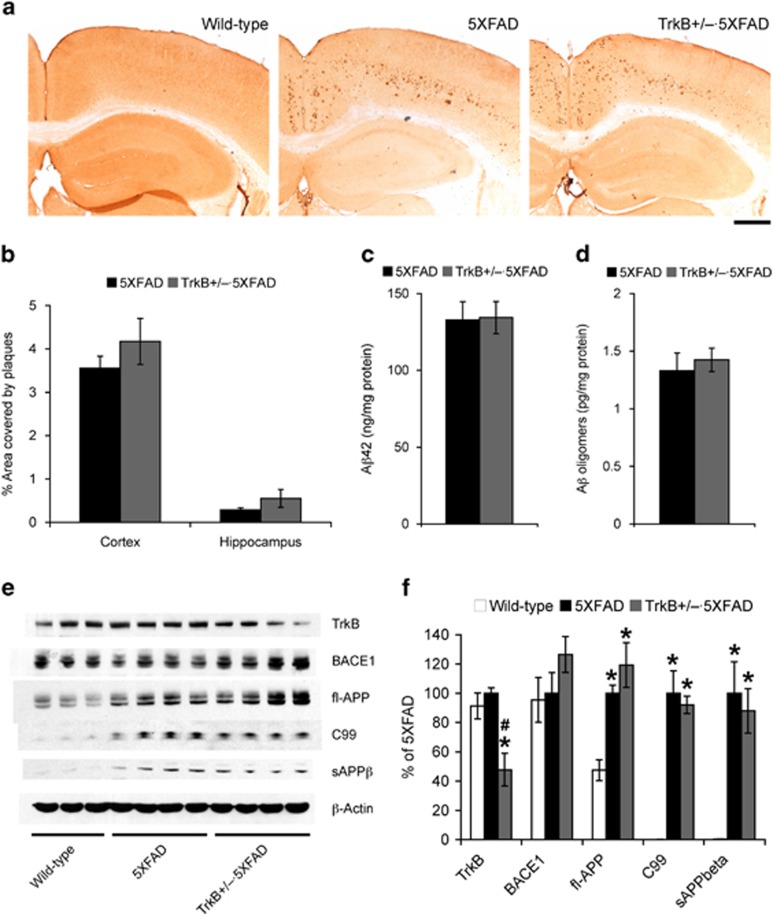
Effects of tropomyosin-related kinase B (TrkB) reduction on amyloid-β (Aβ) accumulation and β-amyloidogenic processing of amyloid precursor protein (APP) in 5XFAD mice. (**a**) Brain sections were immunostained with the 6E10 anti-Aβ antibody. Shown are representative photomicrographs of the hippocampal and cortical regions. Scale bar, 500 μm. (**b**) Percentage area occupied by Aβ deposits in the cerebral cortex and hippocampus was measured for quantification (*n*=4–5 mice per group). (**c**, **d**) Levels of total Aβ42 in guanidine extracts (**c**) and soluble Aβ oligomers in hemibrain samples (**d**) were quantified by sandwich enzyme-linked immunosorbent assays and expressed in nanograms and picograms per milligram of total protein, respectively (*n*=7–12 mice per group). None of Aβ measurements reveals significant differences between TrkB^+/–^·5XFAD and 5XFAD control mice at 4–5 months of age. (**e**) Representative immunoblots of protein extracts from hemibrain homogenates of mice. (**f**) Immunoreactive bands were quantified and expressed as the percentage of 5XFAD control mice (*n*=3–4 mice per group). Although TrkB expression is significantly reduced, levels of BACE1, full-length APP, C99 and sAPPβ are indistinguishable between TrkB^+/–^·5XFAD and 5XFAD control mice at 4–5 months of age (*P<0.05 vs wild type, ^#^*P*<0.05 vs 5XFAD). All data are presented as mean±s.e.m.

To address the mechanisms by which TrkB^+/–^ reduction exacerbated memory deficits in 5XFAD mice, we compared cerebral Aβ accumulation between TrkB^+/–^·5XFAD and 5XFAD control mice (Figure 2). First, Aβ immunostaining with 6E10 antibody revealed that hippocampal and cortical plaque loads in TrkB^+/–^·5XFAD mice at 4–5 months of age were indistinguishable from those in age-matched 5XFAD control mice (Figures 2a and b). Sandwich ELISAs also showed that neither total Aβ42 levels in guanidine-extracted brains (Figure 2c) nor concentrations of soluble Aβ oligomers (Figure 2d) were increased in TrkB^+/–^·5XFAD mouse brains as compared with those of 5XFAD controls.

We further compared the β-amyloidogenic processing of APP with immunoblot analyses of hemibrain samples from these mice (Figures 2e and f). We found no differences between TrkB^+/–^·5XFAD and 5XFAD mice in levels of the β-secretase enzyme BACE1, its substrate full-length APP or direct β-metabolites of APP such as the β-cleaved C-terminal fragment of APP (C99) and soluble ectodomain of APP (sAPPβ). Collectively, TrkB haploinsufficiency, as confirmed by ~50% reduction in protein level, did not affect Aβ production, concentrations or plaque pathology in 5XFAD mice.

### TrkB reduction exacerbates hippocampal signaling aberrations in 5XFAD mice

**Figure 3:**
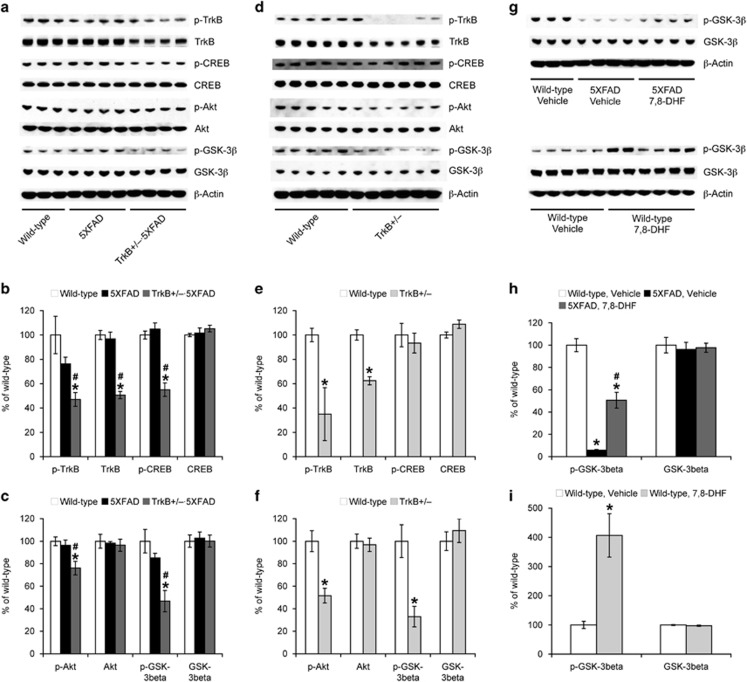
Effects of tropomyosin-related kinase B (TrkB) manipulations on hippocampal signaling aberrations in 5XFAD mice. (**a**, **d**) Representative immunoblots of protein extracts from hippocampal homogenates of mice. (**b**, **c**, **e**, **f**) Immunoreactive bands were quantified and expressed as the percentage of wild-type control mice (*n*=3–6 mice per group). Note that signaling has not yet been significantly affected in 5XFAD mice at 4–5 months of age, whereas impairments of CREB (cAMP response element-binding protein) and Akt/GSK-3β (glycogen synthase kinase-3β) phosphorylation pathways are exacerbated by TrkB reduction in 5XFAD mice (*P<0.05 vs wild type, ^#^*P*<0.05 vs 5XFAD). Moreover, phosphorylation of Akt/GSK-3β, but not that of CREB, in TrkB^+/–^ mice is significantly lower than wild-type controls. (**g**–**i**) Administration of the TrkB agonist 7,8-DHF not only restores deficient GSK-3β phosphorylation in 12-month-old 5XFAD mice but also significantly increases baseline levels of GSK-3β phosphorylation in wild-type mice (*P<0.05 vs wild type/vehicle, ^#^*P*<0.05 vs 5XFAD/vehicle; *n*=3–6 mice per group). All data are presented as mean±s.e.m.

We then compared how TrkB gene reduction affects its downstream signaling pathways in the hippocampus of 5XFAD and wild-type control mice (Figure 3). Levels of phosphorylated TrkB and total TrkB were significantly reduced in TrkB^+/–^·5XFAD (Figures 3a and b) and TrkB^+/–^ (Figures 3d and e) mice as compared with their respective controls (*P*<0.05), demonstrating that haploinsufficiency equivalently impaired hippocampal TrkB signaling on the 5XFAD and wild-type background. Meanwhile, TrkB signaling was normal in 5XFAD control mice at 4–5 months of age. Interestingly, we found that levels of phosphorylated CREB, a key transcription factor that is downstream to TrkB and required for memory consolidation,^49, 50^ were significantly reduced without changes in total CREB in TrkB^+/–^·5XFAD mice consistent with their hippocampal memory deficits, whereas CREB dysfunction was not observed in 5XFAD or TrkB^+/–^ mice.

As TrkB receptor activation is known to result in inactivation of GSK-3β through its phosphorylation at Ser9, we also tested whether TrkB^+/–^ reduction could affect GSK-3β phosphorylation at this inhibitory site. Similar to the alterations in CREB phosphorylation, phosphorylated GSK-3β levels were significantly reduced without changes in total GSK-3β in TrkB^+/–^·5XFAD mice (*P*<0.05) but not in 5XFAD mice (Figures 3a and c). However, levels of phosphorylated GSK-3β were also significantly lower in TrkB^+/–^ mice as compared with those of wild-type controls (*P*<0.05; Figures 3d and f). In accordance with these observations, levels of phosphorylated Akt, an important mediator that induces GSK-3β phosphorylation at Ser9 following TrkB activation, in TrkB^+/–^·5XFAD and TrkB^+/–^ mice were significantly lower than those of their respective controls (*P*<0.05). Therefore, these results suggested that TrkB haploinsufficiency by itself was sufficient to suppress the downstream Akt pathway, thereby resulting in the overactivation of GSK-3β whether it was introduced on the 5XFAD or wild-type background.

To further address the roles of TrkB signaling in GSK-3β regulation, we examined the effects of 7,8-DHF, a small-molecule TrkB agonist, in older 5XFAD mice (Figures 3g and h). We previously reported that 5XFAD mice at 12 months of age show reductions in phosphorylated and total TrkB, whereas administration of 7,8-DHF reverses their deficient hippocampal TrkB signaling and memory deficits in the spontaneous alternation Y-maze.^10^ Consistent with these results, the phosphorylation of GSK-3β at Ser9 was markedly suppressed in 12-month-old 5XFAD mice as compared with wild-type littermate controls (*P*<0.05), while treatments with 7,8-DHF (5 mg kg^−1^, intraperitoneal) partially but significantly restored the reduction of phosphorylated GSK-3β (*P*<0.05). Moreover, we found that overactivation of TrkB receptors with 7,8-DHF in wild-type mice induces a marked increase in the baseline levels of GSK-3β phosphorylation (Figures 3g and i). Together, genetic and pharmacological manipulations revealed that the TrkB/Akt pathway has a critical role in negatively modulating GSK-3β through its Ser9 phosphorylation.

### TrkB reduction exacerbates reductions in hippocampal glutamate receptor expression in 5XFAD mice

**Figure 4:**
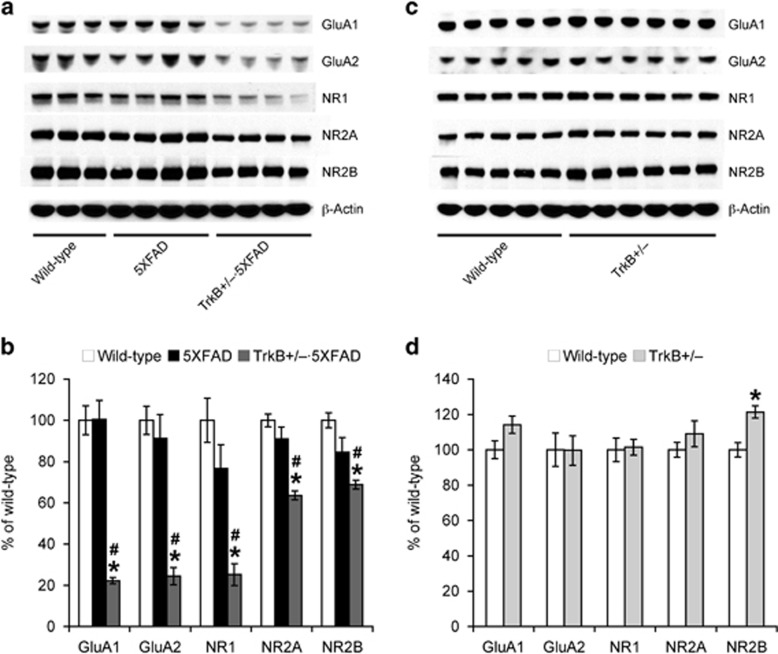
Effects of tropomyosin-related kinase B (TrkB) reduction on hippocampal expression of glutamate receptor subunits in 5XFAD mice. (**a**, **c**) Representative immunoblots of protein extracts from hippocampal homogenates of mice. (**b, d**) Immunoreactive bands were quantified and expressed as the percentage of wild-type control mice (*n*=3–6 mice per group). Note that 5XFAD mice at 4–5 months of age have not yet showed significant changes, while reductions in all of the AMPA and NMDA receptor subunits are observed specifically in TrkB^+/–^·5XFAD mice (*P<0.05 vs wild type, ^#^*P*<0.05 vs 5XFAD). All data are presented as mean±s.e.m.

To address synaptic mechanisms that may account for memory impairments in TrkB^+/–^·5XFAD mice, we performed immunoblot analysis of hippocampal expression of AMPA (GluA1 and GluA2) and NMDA (NR1, NR2A and NR2B) receptor subunits (Figure 4). None of the AMPA or NMDA receptor subunits was significantly reduced in 5XFAD mice at 4–5 months of age as compared with wild-type controls (Figures 4a and b), which was in accordance with our previous findings that basal synaptic transmission and long-term potentiation, a synaptic plasticity model for learning and memory, at the Schaffer collateral-CA1 pathway are still normal in 5XFAD mice at this age.^39^ Notably, we found that expression levels of all the AMPA and NMDA receptor subunits in TrkB^+/–^·5XFAD mice were significantly lower than those of wild-type and 5XFAD mice (*P*<0.05). Furthermore, no difference in glutamate receptor expressions was observed between TrkB^+/–^ and wild-type mice except for the NR2B subunit, which showed a slight but significant increase rather than decrease compared with wild-type controls (*P*<0.05; Figures 4c and d).

## Discussion

It has been reported that levels of BDNF and its receptor TrkB are significantly reduced in brains of patients with mild cognitive impairment or early stages of AD,^1, 2^ while environmental factors that elevate risks for sporadic AD, such as aging, stress and high-fat diet, impair the BDNF/TrkB signaling pathway.^16, 17, 18^ In this study, we used TrkB haploinsufficiency as a model to reproduce the TrkB reduction associated with incipient AD, and investigated the mechanisms by which TrkB reduction may trigger or accelerate AD and memory deficits in the 5XFAD mouse model. We compared AD-like phenotypes of TrkB^+/–^·5XFAD mice with those of 5XFAD control mice at 4–5 months of age, which had only modest Aβ accumulation and still retained normal levels of TrkB signaling and memory function. Our results clearly demonstrated that TrkB^+/–^ reduction in 5XFAD mice exacerbates memory deficits as tested by the hippocampus-dependent spontaneous alternation Y-maze paradigm. In contrast, nest building, an affiliative or social behavior that is displayed by males and females in parental and non-parental settings,^34, 35^ was not impaired in TrkB^+/–^·5XFAD mice, suggesting that the aggravation of AD-like behavioral deficits by TrkB reduction may be memory-specific. Importantly, we found that whereas the degree of TrkB signaling deficiency, as measured by reductions in phosphorylated TrkB, was indistinguishable between TrkB^+/–^·5XFAD and TrkB^+/–^ mice, the memory performance was impaired only in TrkB^+/–^·5XFAD but not in TrkB^+/–^ mice. Therefore, these findings indicate that partial reduction of TrkB signaling caused by haploinsufficiency specifically facilitates AD-associated memory declines without deteriorating baseline memory function. Similar results are reported by testing APP/PS1 mice that are genetically engineered to overexpress a dominant-negative form of truncated TrkB, although there is only a trend toward the facilitation of memory deficits compared with 12-month-old APP/PS1 control mice that have already shown significant memory impairments in the Morris water maze.^51^

What mechanisms can account for memory deficits found in TrkB^+/–^·5XFAD mice? Previous work from our laboratory and others has demonstrated that chronic treatments with the potent and selective TrkB agonist 7,8-DHF alleviate cerebral Aβ accumulation and spatial memory impairments in advanced stages of 5XFAD mice.^10, 11^ Likewise, Rohe *et al.*^14^ report that BDNF treatment reduces Aβ levels in primary cortical neurons of PDAPP mice as well as *in vivo* wild-type mouse brains, whereas Aβ production is facilitated by BDNF deprivation in cultured neurons.^15^ These findings raise the possibility that TrkB^+/–^·5XFAD mice may exhibit memory impairments because of aggravated β-amyloidogenesis. However, we found no difference between TrkB^+/–^·5XFAD and 5XFAD mice in amyloid plaque burdens, total Aβ42 levels and concentrations of soluble Aβ oligomers. Furthermore, TrkB^+/–^ reduction did not affect levels of BACE1 expression or direct β-cleavage metabolites of APP (C99 and sAPPβ) in 5XFAD mouse brains, indicating no changes in the β-amyloidogenic processing of APP. Our results are in agreement with previous studies showing that the overexpression of dominant-negative truncated TrkB and BDNF^+/–^ deficiency exacerbates memory declines in the water maze task but has no effect on cerebral Aβ accumulation in APP/PS1 or 3xTg-AD mice.^51, 52, 53^ Therefore, the investigations using different AD mouse models seem to consistently support the idea that deficient BDNF/TrkB signaling may contribute to the worsening of memory deficits in AD without accelerating β-amyloidosis.

We next compared the changes in memory-associated signaling pathways downstream to TrkB receptors between TrkB^+/–^·5XFAD and TrkB^+/–^ mice. One of the important target molecules is CREB, a transcription factor whose activation is required for long-term potentiation and memory consolidation.^49, 50^ Interestingly, we found that hippocampal CREB dysfunction, as evidenced by a significant reduction in phosphorylated CREB, occurs specifically in TrkB^+/–^·5XFAD mice but not in TrkB^+/–^ mice despite their similar levels of TrkB signaling deficiencies. Moreover, the TrkB/CREB pathway remained normal in 4–5-month-old 5XFAD mice, while Aβ-dependent suppression of CREB function has been reported *in vitro*^54^ and in advanced stages of AD mouse models, including 5XFAD at 8–9 months of age, in close association with their memory deficits.^55, 56^ Taken together, it seems likely that a combination of subthreshold levels of Aβ and dysfunctional TrkB due to heterozygous gene knockout may underlie deficient CREB signaling in TrkB^+/–^·5XFAD mice, consequently leading to the hippocampus-dependent memory impairments.

Another downstream of TrkB is the Akt/GSK-3β pathway, which is known to represent a crucial determinant of the direction of synaptic plasticity in such a way that its overactivation favors long-term depression over long-term potentiation.^57, 58^ In this study, TrkB^+/–^·5XFAD but not 5XFAD mice at 4–5 months of age showed a reduction in phosphorylation of GSK-3β at the inhibitory site (Ser9), reflecting an increased activity of this kinase. Intriguingly, we also found that levels of phosphorylated GSK-3β were significantly lower in TrkB^+/–^ mice as compared with wild-type controls. Consistent with these observations, phosphorylation of Akt, an upstream signaling event that is required to induce GSK-3β phosphorylation at Ser9, was significantly decreased in TrkB^+/–^ mice as well as in TrkB^+/–^·5XFAD mice. Meanwhile, phosphorylated GSK-3β was markedly reduced in older 5XFAD mice (12 months of age) in accordance with our previous data indicating that hippocampal TrkB signaling was impaired in 5XFAD mice at this age.^10^ Importantly, we found that pharmacological activation of TrkB receptors with 7,8-DHF not only restores reduced GSK-3β phosphorylation in older 5XFAD mice but also increases baseline levels of GSK-3β phosphorylation in wild-type mice. Taken collectively, our results demonstrate a direct role of the TrkB/Akt pathway in mediating phosphorylation of GSK-3β at Ser9, providing *in vivo* evidence that deficient TrkB signaling by itself (that is, independent of Aβ) may be sufficient to induce the overactivation of GSK-3β as a consequence of reduction of Ser9 phosphorylation. Although TrkB^+/–^ mice with decreased GSK-3β phosphorylation showed normal learning and memory, our results do not necessarily rule out the possibility that long-term depression-favoring properties of GSK-3β overactivation^57, 58^ in conjunction with subthreshold levels of Aβ accumulation may contribute to the facilitation of memory deficits found in TrkB^+/–^·5XFAD mice. Moreover, given that the amyloid model of 5XFAD used in this study lacks tau pathology, further study will be needed to determine whether TrkB reduction may lead to the acceleration of neurofibrillary tangle formation in relevant tau transgenic animal models.

Finally, we explored whether hippocampal glutamate receptor expression could be altered in TrkB^+/–^·5XFAD mice as associated with their memory impairments. Remarkably, our results demonstrated that TrkB haploinsufficiency significantly lowers levels of both AMPA (GluA1 and GluA2) and NMDA (NR1, NR2A and NR2B) receptor subunits in 5XFAD mice at 4–5 months of age. In contrast, none of these glutamate receptor subunits was reduced in 5XFAD or TrkB^+/–^ mice that showed normal memory function. Previous reports from our laboratory and others have shown that 5XFAD mice start to show CA1 synaptic dysfunctions, as assessed by reductions in baseline excitatory transmission (mainly AMPA receptor-mediated responses) and long-term potentiation (a model of NMDA receptor-dependent synaptic plasticity), at 6 months of age in accordance with the onset of hippocampal memory declines.^11, 26, 38, 39, 40, 41, 42, 43, 44^ Moreover, biochemical examinations also reveal that hippocampal expression of AMPA and NMDA receptor subunits is significantly reduced in 6-month-old 5XFAD mice.^42, 43^ Therefore, it seems reasonable to conceive that TrkB reduction may accelerate progressive Aβ-dependent deterioration of AMPA and NMDA receptor expression in the hippocampus of 5XFAD mice without affecting β-amyloidosis, which consequently leads to the exacerbation of learning and memory impairments.

In conclusion, the present investigation provides experimental evidence that deficient TrkB signaling during incipient stages of AD may not affect β-amyloidosis but exacerbate the manifestation of Aβ-dependent deteriorations including hippocampal memory declines, CREB dysfunction and reductions in AMPA/NMDA receptor expression. Meanwhile, it is strongly suggested that a reduction of TrkB may directly modulate the Akt/GSK-3β pathway independent of Aβ, thereby contributing to the overactivation of GSK-3β in AD.
